# Regulation of Immunogen Processing: Signal Sequences and Their Application for the New Generation of DNA-Vaccines

**Published:** 2010-04

**Authors:** E.S. Starodubova, M.G. Isaguliants, V.L. Karpov

**Affiliations:** Engelhardt Institute of Molecular Biology, Russian Academy of Science; Karolinska Institutet, Sweden; Ivanovsky Institute of Virology, Russian Academy of Medical Science

**Keywords:** DNA-vaccines, МНС-I, MHC-II, antigen presentation

## Abstract

Immunization with naked genes (DNA–immunization) is a perspective modern approach to prophylactic as well as therapeutic vaccination against pathogens, as well as cancer and allergy. A panel of DNA immunogens has been developed, some are already in the clinical trials. However, the immunogenicity of DNA vaccines, specifically of those applied to humans, needs a considerable improvement. There are several approaches to increase DNA vaccine immunogenicity. One approach implies the modifications of the encoded immunogen that change its processing and presentation, and thus the overall pattern of anti–immunogen response. For this, eukaryotic expression vectors are constructed that encode the chimeric proteins composed of the immunogen and specialized targeting or signal sequences. The review describes a number of signals that if fused to immunogen, target it into the predefined subcellular compartments. The review gives examples of their application for DNA–immunization.

## INTRODUCTION

**Fig. 1:**
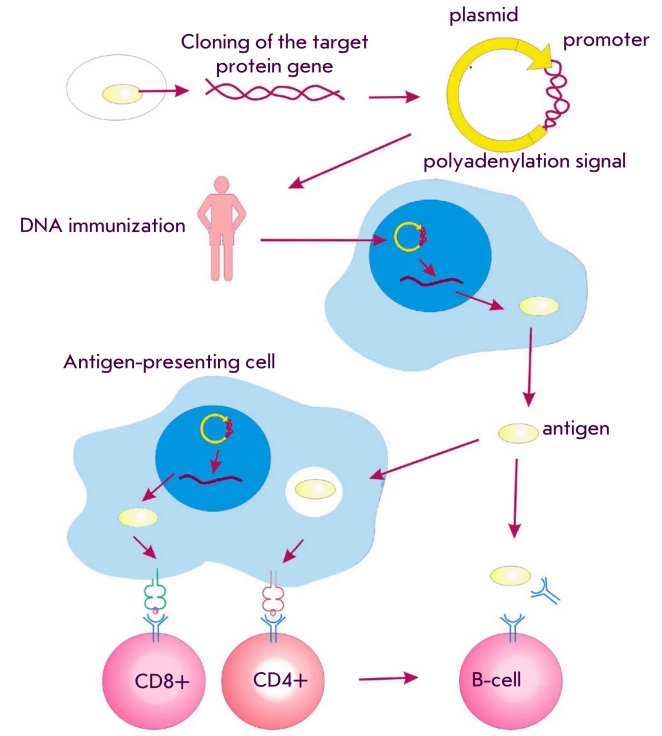
DNA-immunization

One of the most promising vaccine types today are DNA–vaccines. In its simplest form, a DNA–vaccine is a plasmid containing a gene of the pathogenic protein and the elements needed to transcribe this gene in mammalian cells. This DNA is introduced into mammalian cells during immunization. It is then transcribed, and the encoded antigen is synthesized initiating an immune response (Fig. 1). Unlike protein–based vaccines, DNA–vaccines based on microbial genes and tumor antigens have the advantage of synthesizing the specific antigen in the host’s organism, where it is processed correctly to induce an immune response of the desired specificity. This approach is promising because of the simplicity and low cost of the production and transportation of DNA–vaccines as compared to the traditional vaccines. Moreover, gene engineering allows an easy modification of DNA–immunogens; new antigens can be designed with properties predicted by * in silico * studies. The use of DNA–vaccines causes some anxiety because of the possibility that the genetic material gets inserted into the host genome (insertional mutagenesis). However, the probability of this event is extremely low. It is in the range of around 1–7 insertions per 150,000 nuclei, which is lower than the rate of the natural insertion mutation by a factor of 1,000 [1].

DNA–vaccines are used to induce a protective immune response against various infections in small animals (rodents) and in larger species [2–4]. Series of trials of prophylactic and therapeutic DNA–vaccines against various human pathogens, including HIV–1 and HCV, have been performed [5–7]. However, the immunogenicity of genetic vaccines needs improvement, especially for human applications [8–10]. Various approaches are being used in order to increase the efficiency of DNA–vaccines [11–13]. They include the development of novel methods of DNA–vaccine administration (electroporation has become increasingly popular in the recent years); supplementing vaccine formulation with cytokines and/or chemokines or their genes [14] ; optimizing plasmid vectors by selecting more effective gene promoters and regulatory elements [15] ; and modulating plasmid CpG content [14]. The DNA–immunogen is also modified: the coding sequence of immunogen is often changed to increase the expression [16]. One of the most promising approaches for modifying the immunogen is to alter its processing and presentation pathway [17]. Such re–direction can be achieved by “labeling” the protein with specialized signal sequences.

This review focuses on the signals directing protein into various cellular compartments and their use for DNA–vaccine design. In order to be recognized by the immune system, an antigen must be processed and presented on the surface of a cell by the molecules of the major histocompatibility complex (MHC). There are two main classes of these molecules: MHC class I (MHC–I) and MHC class II (MHC–II). In order to bind to the molecules of either class, the protein encoded by DNA–immunogen must go through antigen processing in the specialized cell compartments (Table 1). Endogenous proteins are degraded in the proteasome and are presented in a complex with МНС –I on the cell surface, where they can be recognized by the receptors of cytotoxic CD8+ T–cells (CTL), which then initiate a cytotoxic immune response [18]. Exogenous proteins are hydrolyzed by proteases in the lysosome, antigen fragments generated by processing are bound to MHC–II molecules and recognized by the CD4–receptors of T–helper cells, which facilitate cellular as well as humoral responses [19]. Thus, it is evident that the processing pathway of the immunogen determines the type of immune response it induces.

## Presentation of a DNA-encoded antigen via the MHC-I pathway

**Fig. 2:**
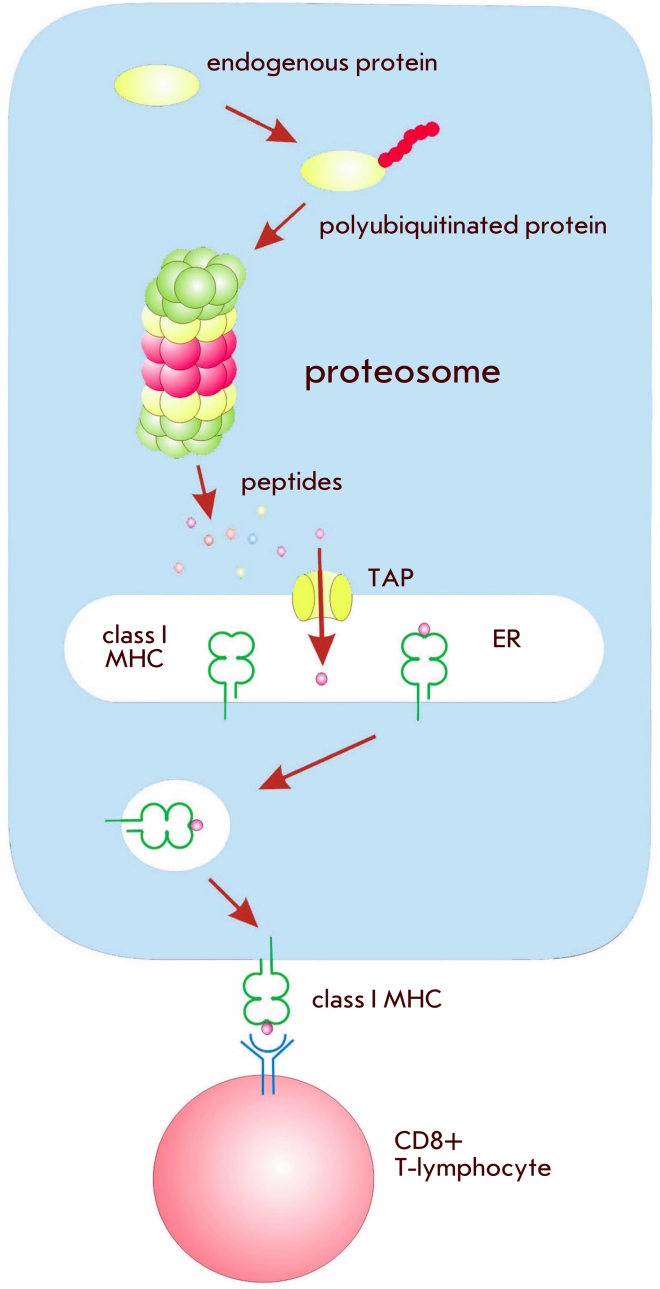
Antigen processing during presentation via the MHC-I pathway

As has been mentioned earlier, processing of an antigen via the MHC–I pathway results in a CTL–response. This process involves several steps (Fig. 2). The protein is synthesized in the cell and then broken into small peptides in the proteasome, after which these fragments are taken up by the transporter–proteins associated with antigen processing (TAP). These proteins guide the peptides into the endoplasmic reticulum (ER), where they can bind to MHC–I molecules [18].

The peptide–MHC–I complex is transported to the cell surface to be recognized by CD8+ T–cells (CTL), manifesting the initiation of a cellular response. This is why increasing the amount of the protein that is transported into the proteasome or ER should in principle increase its presentation via MHC–I pathway, hence, the availability on the cell surface resulting in an enhancement of the cellular response.

## Proteasome-mediated mechanism

To be degraded by the proteasome, proteins must bear a specific signal – a chain of ubiquitin molecules (Ub), small polypeptides consisting of 76 amino acid residues. The eukaryotic cell has a specialized group of enzymes that recognize protein substrates and covalently attach polyubiquitin to these substrates. This enzymatic group is called the ubiquitin–conjugating system [20].

This system recognizes various proteasome degradation signals. Signals can represent a specific amino acid sequence, a specific pattern of protein phosphorylation, or alterations in the protein structure, often missfolding. Several degradation signals with a characteristic amino acid sequence have been described. The first signal discovered was the N–degron. This is a specific first amino acid residue in the protein that serves as a substrate for the complex of cellular enzymes responsible for labeling the protein with a polyubiquitine chain [21]. Amino acids differ in their capacity to be recognized by the ubiquitinating enzymes (Table 2). There are also other degradation signals, such as the PEST–sequence [22] and the Destruction Box [23], often found in the short–lived cellular proteins (Table 2).The nature of the first amino acid residue in the protein is, thus, one of the main protein features determining its accumulation.

**Table 1:** Antigen processing pathways and types of immune responses.

Antigen localization	Main processing compartment	Antigen presenting complex	Recognition cells of the immune system	Stimulated immune response
Inside the cell	Proteosome	MHC-I	CD8+	Cytotoxic
Outside the cell	Lysosome	MHC-II	CD4+	Cellular, humoral

## Ubiquitin-dependent mechanism

The main focus of the researchers has been on the antigen (re)targeting into proteasome via the ubiquitin–dependent mechanism, thought to provide for antigen processing and presentation in complex with MHC–I and an enhanced antigen–specific CTL–response [25–28]. This (re)direction can be achieved by cloning an Ub–encoding sequence onto the 5 ´ –terminus of the target gene and adding a destabilizing N–terminal residue after the Ub, which makes an antigen a better proteasome substrate. In the cell, Ub is cleaved off in a posttranslational modification of the chimeric protein catalyzed by the C–terminal ubiquitin hydrolase, thus exposing the N–degron. The HIV–1 nef was modified in this way to generate a Ub–Arg–Nef, which led to the improved immunogenicity of nef in mice [29]. Fusion with ubiquitin was used for immunization with HIV–1 gene expression libraries. All of the ORFs (open reading frames) of HIV–1 encoded by 32 plasmids were modified by the addition of an ubiquitin encoding sequence. After a single immunization using gene gun, this library stimulated a strong T–cell response against all 32 antigens. This response was registered as an enhanced CTL–activity, IFN– γ + production by CD8+ T–cells and HLA–tetramer binding [30]. Addition of ubiquitin to the N–terminus of a synthetic protein consisting of HIV CTL–epitopes also resulted in the increase of immunogenicity of this prototype DNA–vaccine [31].

An incorrect protein folding can also act as the proteasome degradation signal. This was used to increase the immunogenicity of the influenza virus proteins M1 and NS1. Unstable variants of M1 and NS1 were constructed by disrupting their alpha–helical regions via introduction of short (foreign) amino acid sequences. Immunization by the genes of the destructured M1 and NS1 resulted in a much stronger CTL–response than that induced by the original genes [32].

However, for some viral proteins, fusion with the proteasome–targeting signals did not result in an increased degradation [27, 33]. The HIV–1 Gag protein modified by ubiquitination and by a destabilizing N–terminal arginine residue (Ub–R–Gag) showed only a slight increase in the degradation rate. Effective destabilization of this protein required an insertion of the additional exposed lysine residue eK (Ub–R–eK–Gag). The Ub–R–eK–Gag chimera was effectively directed into the proteasome, which increased the presentation of MHC–I–antigen peptide complexes on the cell surface. However, this did not significantly increase the anti–Gag CTL–response in mouse immunization [34]. Also, no enhancement in immunogenic performance was observed after a similar modification of DNA–immunogen expressing nucleocapsid (core) protein of Hepatitis C virus (HCV). HCV core genes carrying cleavable, as well as uncleavable ubiquitin residues with N–stabilizing or N–destabilizing amino acid residues, were equally poor immunogens [35]. Other (viral) models were described for which an increase in the proteasomal degradation did not result in an increased protective immunity [34].

## Ubiquitin-independent mechanism

Notably, some proteins do not require ubiquitin for degradation [36]. The first such protein to be discovered was ornithine decarboxylase (ODC) [37]. Its degradation is ATP– and antizyme–protein–dependent. The C–terminus of the antizyme binds to the N–terminal region of ODC, directing it to the proteasome while the antizyme is released. In addition to the antizyme–binding site on the N–terminus, ODC contains also a C–terminal PEST–signal [38]. Experiments with deletion mutants of ODC have shown that the minimal signal required for the rapid degradation of ODC in the proteasome is a 37–residue C–terminal stretch of amino acids [39]. It was demonstrated that this region is required for binding of ODC to the proteasome.

The ubiquitin–conjugating system is a multi–stage mechanism with a complex regulation. The use of protein degradation signals, which direct proteins to the proteasome via an ubiquitin–independent mechanism, circumvents the effects of a multitude of factors and thus provides a more straight–forward way of proteasome targeting. Fusion of HIV–1 reverse transcriptase with the minimal proteasome–targeting signals of ODC represented by two short amino acid sequences at the ODC C–terminus led to an accelerated degradation and an increased immunogenicity of the chimeric gene in mice as compared to the original gene [40]. This modification was also successful when applied to the weakly immunogenic reverse transcriptase of drug–resistant HIV–1 [41] helping to enhance both cellular and antibody immune responses against the mutant enzyme form [42].

## ER–mediated mechanism

Processing of an antigen via the МНС –I pathway involves the endoplasmic reticulum (ER). That is why increasing the antigen’s affinity towards ER can improve the immunogenic performance of the antigen. The Ca2+–binding protein calreticulin (CRT) is abundant in ER, where it is associated with the components involved in the presentation of the antigen via the МНС –I pathway [43– 45]. Fusion of calreticulin to the tumor antigens was used to improve the T–cell immune response against tumor cells. A DNA–vaccine that encoded a fusion of CRT with Е 7 protein of human papilloma virus 16 (HPV–16) was created. Mice immunized by this DNA–immunogen exhibited a significant increase in the population of the E7–specific CD8+ T–cells and in their lytic activity against E7–expressing tumors [46, 47]. A fusion–protein of CRT with another HPV–16 protein E6 also improved the antigen–specific CD8+ T–cell immune response in mice [48].

Altered localization of Е 7 HPV–16 protein associated with an increased affinity for ER was also achieved by a different strategy that involved antigen fusion with the extracellular domain of the Fms–like tyrosine kinase 3 ligand (FL) [49]. The E7 gene fusion introduced subcutaneously by the gene gun technique led to a considerably increased capacity of E7 to activate specific CD8+ T–cells compared to the unmodified Е 7 gene. * In vitro * studies showed that 293 cell lines transfected with FL–E7 DNA presented Е 7 antigen in complex with MHC class I molecules more effectively than cells transfected with the original E7 gene. The FL–E7 chimera potently activated CD8+ T–cells; anti–tumor effect was dependent on the CD4+ Т –cells [49]. Another successful case of enhancing the immune response by manipulating the ER–signals was reported for the envelope protein E2 of the hepatitis C virus. The effect was achieved by duplication of the ER–localization sequence, which promoted the accumulation and subsequent release of E2 from the endoplasmic reticulum [50].

## Presentationof a DNA-encoded antigen via the MHC-II pathway

**Fig. 3:**
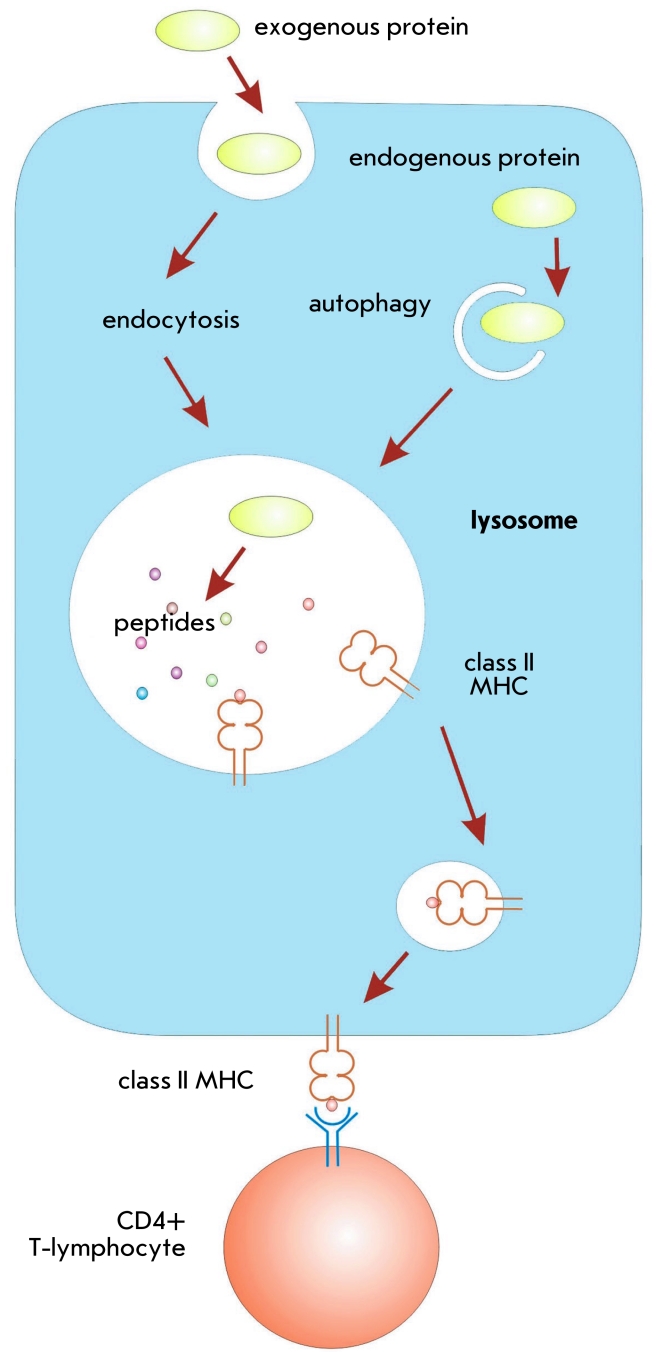
Antigen processing during presentation via the MHC-II pathway

Peptides to be bound to MHC–II are mainly of exogenous origin and are captured by endocytosis to be directed into the lysosomes (Fig. 3). However, it has been demonstrated that some intracellular proteins can be presented by MHC–II as a result of autophagy [51]. Such proteins are transported into the lysosome via the chaperone–mediated transfer carried out by a transport–protein; by the engulfment of cytoplasm by the lysosome membrane, or by the formation of double–membrane autophagosomes [51–55]. After transporting to the lysosomes, the antigen is cleaved by the acidic proteases and the resulting peptides are loaded onto the MHC–II molecules and brought to the cell surface. On the cell surface, these complexes are recognized by the CD4+ T–cell receptors [56]. This leads to the stimulation of cellular (Th1–type) and humoral (Th2–type) immunity.

Traditional therapeutic vaccinations with soluble protein antigens aim to recruit CD4+ T–cells. The relatively weak response of CD4+ T–cells is one of the weak points of DNA–vaccines today. Clinical trials have also shown that the current generation of DNA–vaccines cannot induce a strong antibody response [57]. Therefore, targeting of antigen presentation into the MHC–II pathway in order to activate CD4+ Т –cells seems especially advantageous. Such targeting can be achieved artificially by supplementing immunogen with lysosome localization signals.

The DNA–antigen can be specifically (re)directed into lysosomes using protein sorting signals. Such intracellular sorting signals can be found in the cytoplasm–terminal regions of the transmembrane and lysosome–associated proteins [24, 58, 59]. Most of them are short amino acid sequences and can be divided into the tyrosine– and dileucine–bearing sequences [24, 60]. Tyrosine–bearing signals have a consensus motif NPXY or YXX (where X is any amino acid, and Ø is an amino acid with a large hydrophobic side–chain). The consensus sequence of the dileucine signals is (DE)XXXL(LI) or DXXLL. These signals are recognized by the adaptor protein AP or by related complexes and are then directed into the trans–Golgi, to the plasmatic membrane, and further into the endosomes. There are other motifs, such as a cluster of acidic amino acids and the NPFSD sequence [58]. A number of cellular proteins are directed into lysosomes due to the presence of a phosphorylated mannose residue attached to the consensus sequence NX(ST) [61–63]. There are also signals that seem to direct cytoplasmic proteins into the lysosome via an autophagosome mechanism [64].

## Lysosome-mediated mechanism

It has been shown that tyrosine– and dileucine signals can effectively direct heterologous proteins into the lysosome [19, 65, 66]. The most actively used signals targeting to MHC–II presentation are those of the lysosome–associated membrane protein 1(LAMP–1) [67]; invariant chain (Ii) [65], and the AP3– binding motif of the lysosome protein LIMP II [66].

## LAMP-1 signal

Sorting signal of the lysosome–associated protein 1 (LAMP–1) targets the antigen to processing via the MHC–II presentation pathway and enhances its presentation to CD4+ Т –cells, as has been shown in * in vitro* experiments. Mouse immunization experiments have demonstrated that LAMP–1gene chimeras induced stronger lymphoproliferative activity, CTL–activity and higher antibody titers as compared to the nonmodified DNA–immunogens. An increase in the Th2–type immune response of CD4+ Т –cells in response to the LAMP–1 fusions was shown after immunization with DNA encoding LAMP–1 fusions of HIV–1 gp160– and p55gag [68, 69]. A LAMP/gag DNA–vaccine stimulated prolonged B–, CD4+ and CD8+ T–cell responses, while an immune response caused by the injection of a nonmodified Gag gene rapidly receded [70]. Another successfully redirected cytoplasmic protein was the nucleocapsid protein of the coronavirus SARS (sarsN). Immunization of mice with DNA encoding the LAMP–1–sarsN chimera led to a balanced specific IFN–γ and IL–4 production and strong CTL–response against sarsN [66]. Also, fusion of HIV–1 reverse transcriptase with LAMP–1 improved the immunogenicity of a prototype DNA–vaccine against a drug–resistant virus. A strong immune response of the mixed Th1/Th2–type was raised both against the wild and drug–resistant HIV–1 reverse transcriptases, circumventing tolerance of the immune system towards this conserved retroviral antigen [71].

Fusion with LAMP–1 increased the immunogenicity of DNA immunogens encoding flavivirus envelope proteins. In a candidate vaccine against Dengue virus type 2 based on the DNA encoding the premembrane (preM) and envelope (E), the transmembrane and cytoplasmic domains of E were replaced by similar domains of LAMP–1 [72;]. LAMP–1/preM–E chimera exhibited a characteristic granular cytoplasmic staining that indicated co–localization with the endogenous LAMP–1, MHC–II, and H2–M proteins that was not observed in the case of the nonmodified antigen. Mice immunized with the gene of the LAMP–1/preM–E chimera exhibited a much higher level of neutralizing antibodies than the controls that received the parental preM–E gene. A similar prototype DNA–vaccine was designed against the West Nile virus. In this case, the premembrane and envelope (WN preM–E) coding sequences were fused to the sequences encoding the transmembrane and cytoplasmic domains of LAMP–1 [73]. Mice immunized by the gene of the WN LAMP–1/preM–E chimera responded by a long–lasting production of high titers of neutralizing antibodies, while DNA encoding the original antigen induced a short–termed low–titer antibody response. Altogether, these results provide a basis for creating a panel of effective DNA–vaccines against flaviviruses.

Introduction of the HPV–16 Е 7 protein gene, fused to the sequence encoding LAMP–1, also increased the Th2–type immune response [74]. Introduction of a secretory variant of E7/LAMP–1 in the form of a DNA–chimeric recombinant virus induced a strong anti–tumor immune response, which prevented the formation of tumors and reduced the size of the ones already present [75].

**Table 2:** Signals for proteosome degradation with specific amino acid sequences

Name of signal	Amino acid sequence
N-degron	N-terminal amino acid (recognizing enzyme): Destabilizing – F, L, W, Y, I, R, K, H, A, S, T, G (Е3 -ligase); N,Q (N- N,Q (N-terminal hydrolase); D,E,C (Arg-t-RNA-transferase) Stabilizing – M, S, G, V (no recognizing enzymes)
PEST- sequence	Sequence rich in proline (P), glutamic acid (E), serine (S), and threonine (T)
Destruction box	R-A/T-A-L-G-X-I/V-G/T-N

There are, however, few unsuccessful cases of applying this modification to the cytoplasmic proteins, as in the case of the nucleocapsid protein of HCV and of p53 [35, 76]. A plasmid was constructed expressing a chimeric fusion protein of HCV nuclecapsid protein with signal– and C–terminal LAMP–1 sequences. Immunization of mice with this construct did not lead to any detectable antibody response or cell proliferation and induced only weak CTL–activity [35]. Thus, direction into the lysosome by fusing the immunogen to LAMP–1 does not necessarily ensure an enhancement of the Th–2 type immune response.

## Invariable chain signal

MHC–II molecules require transportation into the lysosome compartment. This transport involves the invariant chain of MHC class II molecules (Ii) [77]. Two sorting signals were found in the cytoplasmic domain of Ii [65, 77, 78]. It was shown that endogenously synthesized proteins, normally not presented via the MHC–II pathway, can be effectively presented by MHC–II if fused to Ii [79]. Numerous experiments in the animal models demonstrated that fusion of the recombinant antigens to Ii can enhance, broaden, and prolong the protective immune response to the resulting chimeric DNA–vaccines. * In vitro * and * in vivo * experiments had shown that immunogens based on an adenovirus expressing fusion of Ii with the glycoprotein of lymphocytic choriomeningitis virus (LCMV) had an increased ability to stimulate LCMV–specific CD4+ and CD8+ T–cells. Moreover, mice that had been immunized by this plasmid only once were resistant to the infection by the lethal LCMV dose [80].

This approach is also effective for immunization of larger species. A DNA–construct was made encoding the major surface protein 1a of * Anaplasma marginale * fused with the lysosome–targeting motif of the bovine Ii, which directed the chimera into the lysosome compartment [81]. A single dose of this plasmid effectively stimulated an immune response seen as a potent proliferation of IFN– γ +/CD4+ T–cells and production of specific IgG. A single injection of this construct induced antigen specific memory cells, which formed the basis for an accelerated response to repeated doses of the antigen [81].

## Direction into autophagosomes

The precise mechanisms behind autophagy are yet unknown despite the intensive ongoing studies. With regard to antigen retargeting, it was shown that fusion of the autophagosome–associated protein Atg8/LC3 with the influenza virus matrix protein 1 leads to a considerable increase in the MHC class II presentation and in the M1–specific response of CD4+ T–cells [82]. This confirms that autophagy constantly and effectively directs cytoplasmic proteins into presentation via the MHC class II pathway, where they can be used to stimulate a Т –helper response.

## Secretory direction

An effective approach to the induction of a T–helper immune response is targeting of proteins for secretion into the extracellular environment. Fusion of HIV–1 Gag and E proteins to the secretory chemokine MCP3 directed these viral proteins into the secretory pathway. Chimeric genes induced an effective production of anti–HIV–1 antibodies in macaques. Macaques immunized with the chimeras and infected with a pathogenic SIVmac251 had lower viral loads than the infected na ï ve animals [83]. This is an example of the conversion of an endogenous antigen into the exogenous, for further capture by endocytosis and transfer to the lysosomes. This pathway, as well as the endogenous lysosome (re)targeting, enhances antigen presentation in complex with the MHC class II molecules and can considerably increase the immunogenicity of DNA–vaccines.

## CONCLUSIONS

Today, mankind faces an acute problem of creating vaccines against such hazardous diseases as hepatitis C, the immunodeficiency caused by HIV–1, and cancer. DNA–vaccine technology opens a wide range of possibilities for creating effective vaccines, one such is through immunogen (re)targeting. In this paper, we reviewed a number of signal sequences that can be introduced into the immunogens to direct them into a predetermined processing and presentation pathway, usually different from the one they would naturally take. Direction of an antigen into the MHC–I presentation pathway would enhance the cytotoxic Т –cell response, while direction onto the MHC–II pathway would activate T–helper cells and stimulate both cellular and humoral responses. Overall, the use of signal sequences to control and guide immunogen presentation can increase the immunogenic potential of the existing DNA–immunogens and help to create new effective prophylactic and therapeutic vaccines for diverse human applications.

## ABBREVIATIONS

ER: endoplasmic reticulum
Ub: ubiquitin
HCV: human hepatitis C virus
ODC: Ornithine decarboxylase
RT: HIV-1 reverse transcriptase
CRT: Ca^2+^-binding protein calreticulin
HVP-16: human papilloma virus 16
LAMP-1: lysosome-associated protein 1
sarsN: nucleocapsid SARS coronavirus protein
LCMV: lymphocytic choriomeningitis virus
MHC I: major histocompatibility complex class I
MHC II: major histocompatibility complex class II
